# Knockdown of miR-214 Promotes Apoptosis and Inhibits Cell Proliferation in Nasopharyngeal Carcinoma

**DOI:** 10.1371/journal.pone.0086149

**Published:** 2014-01-21

**Authors:** Zi-Chen Zhang, Yang-Yang Li, Hai-Yun Wang, Sha Fu, Xiao-Pai Wang, Mu-Sheng Zeng, Yi-Xin Zeng, Jian-Yong Shao

**Affiliations:** 1 Department of Molecular Diagnostics, Sun Yat-sen University Cancer Center, State Key Laboratory of Oncology in South China, Collaborative Innovation Center for Cancer Medicine, Guangzhou, China; 2 Department of Experimental Research, Sun Yat-sen University Cancer Center, State Key Laboratory of Oncology in South China, Collaborative Innovation Center for Cancer Medicine, Guangzhou, China; Central South University, China

## Abstract

MicroRNA-214 (MiR-214) is aberrantly expressed in several human tumors such as ovarian cancer and breast cancer. However, the role of miR-214 in nasopharyngeal carcinoma (NPC) is still unknown. In this study, we report that miR-214 was overexpressed in NPC cell lines and tissues. Silencing of miR-214 by LNA-antimiR-214 in NPC cells resulted in promoting apoptosis and suppressing cell proliferation *in vitro*, and suppressed tumor growth in nude mice *in vivo*. Luciferase reporter assay was performed to identify Bim as a direct target of miR-214. Furthermore, this study showed that low Bim expression in NPC tissues correlated with poor survival of NPC patients. Taken together, our findings suggest that miR-214 plays an important role in NPC carcinogenesis.

## Introduction

MicroRNAs (miRNAs) are small, endogenous and non-coding RNAs, which modulate gene expression by binding to the 3′ untranslated region (3′ UTR) of target mRNA, and promoting RNA degradation, inhibiting mRNA translation, and affecting transcription [1]. Growing evidence has suggested that miRNAs play important roles in various biological processes, such as cell proliferation, differentiation and programmed cell death [1], [2]. Recent studies have shown that miRNA mutations or abnormal expression in many human cancers, indicating that they may function as tumour suppressor genes (TSG) and oncogenes [3], [4], [5], [6]. They have the potential to regulate various critical biological processes, including the differentiation, apoptosis, proliferation and metastasis of tumor cells [7], [8], [9].

MiR-214 is upregulated in several human tumors, such as ovarian cancer [10], gastric cancer [11], Sézary syndrome [12] and melanoma [13], but downregulation in cervical cancer [14], [15], [16], pancreatic cancer [17], hepatocellular carcinoma [18], [19] and breast cancer [20]. In addition, miR-214 induces cell survival and cisplatin resistance by binding to 3′-UTR of PTEN leading to inhibition of PTEN translation and activation of Akt pathway [21]. MiR-214 promoted survival of pancreatic cancer cells as well as GEM resistance, which might be related to the poor response to chemotherapy in pancreatic cancer patients [17].

However, the role of miR-214 in nasopharyngeal carcinoma (NPC) is still unknown. Thus, we investigated the expression level of miR-214 in NPC compared to normal nasopharyngeal epithelium, and further studied the possible function of miR-214 in the NPC cell lines. The results showed that miR-214 was overexpressed in human NPC and knockdown of miR-214 increased apoptosis and suppressed cell growth *in vitro* as well as *in vivo*. Furthermore, we identified Bim (Bcl-2-interacting mediator of cell death) as a direct target of miR-214.

## Results

### MiR-214 is overexpressed in NPC tissues and cell lines

**Figure 1:**
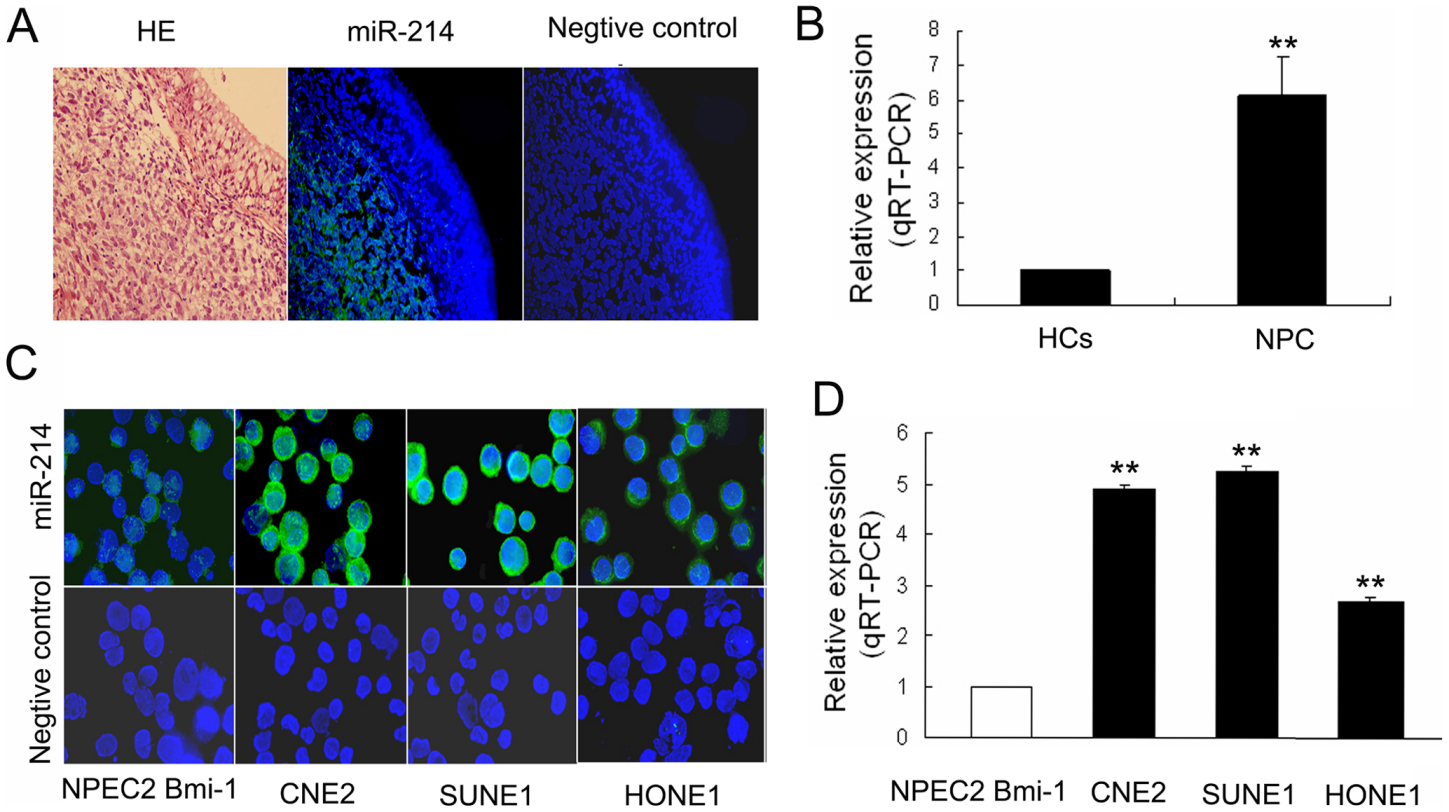
Expression of miR-214 in NPC tissues and NPC cell lines. (A) Representative picture of fluorescence density for miR-214 expression in 5 NPC patients compared to adjacent normal tissues (400× magnification). MiR-214 positive signals detected by FISH are in green, which is upregulated in NPC tumor cells compared to the adjacent epithelial cells. (B) Relative quantitative analysis by qRT-PCR showed that expression of miR-214 is significantly higher in NPC tissues than healthy control (*P* = 0.001). (C) FISH detection of miR-214 in three NPC cell lines (CNE2, SUNE1, and HONE1) and one non-tumorigenic epithelial cell line (NPEC2 Bmi-1). MiR-214 positive signals are visualized in green (D). Relative qRT-PCR analysis shows that CNE2, SUNE1, and HONE1 cells express higher levels of miR-214 compared with NPEC2 Bmi-1 cells. All data are shown as mean ± SD of triplicate experiments (** *P*<0.01). Abbreviations: NPC, Nasopharyngeal carcinoma; NATs, normal adjacent tissues, qRT-PCR, quantitative RT-PCR, SD, standard deviation.

We performed fluorescence in situ hybridization (FISH) on 5 NPC patients and 5 healthy controls (HCs) to examine the expression of miR-214. The representative pictures were shown in Figure 1A. Obviously, the overexpression of miR-214 was observed in NPC tissues compared to normal adjacent tissue (NATs). Also, quantitative RT-PCR (qRT-PCR) was utilized to test the miR-214 level in NPC patients and HCs. The miR-214 showed higher expression in NPC patients relative to HCs (Figure 1B, *P* = 0.001, n = 5). Similarly, FISH and qRT-PCR were used to measure miR-214 levels in three NPC cell lines (CNE2, SUNE1 and HONE1) and one immortalized nasopharyngeal epithelial cell line (NPEC2 Bmi-1), respectively. The results were shown in Figure 1C–D. These findings suggested that miR-214 was overexpressed in NPC cell lines and tissues.

### Knockdown of miR-214 induces apoptosis *in vitro*

**Figure 2:**
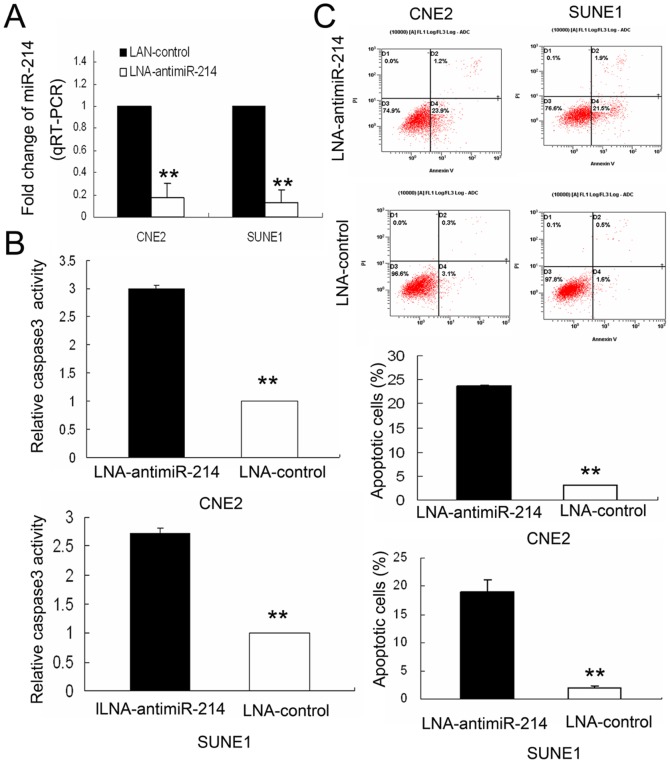
Silencing of miR-214 induces apoptosis in NPC cells. (A) Forty-eight hours post-transfection, miR-214 expression levels (normalized to *U6 RNA*) were significantly decreased by 82% (*P*<0.01) in CNE2/LNA-antimiR-214 and 87.7% (*P*<0.01) in SUNE1/LNA-antimiR-214 cells, relative to the LNA-control. (B) Forty-eight hours post-transfection, Caspase 3 activity in the cells were measured by spectrophotometry. The relative activity of Caspase 3 was significantly increased in CNE2/LNA-antimiR-214 cells and SUNE1/LNA-antimiR-214 cells. (C) Forty-eight hours post-transfection, apoptosis was determined by flow cytometric detection of Annexin-V-FITC-positive/PI-negative cells. Bars are shown as the mean ± SD of cells with Annexin-V-FITC-positive and PI-negative. All data are shown as mean ± SD of triplicate experiments. ** *P*<0.01.

CNE2 and SUNE1 cells were transfected with LNA-antimiR-214 or LNA-control. QRT-PCR was performed to confirm miR-214 expression of CNE2 and SUNE1 cells transfected with LNA-antimiR-214 (50 nM) after 48 hours transfection. The decreased levels of miR-214 in the two cell lines were depicted compared to these cells transfected with LAN-control (Figure 2A).

To investigate the effect of miR-214 silencing on the apoptosis of NPC cells (CNE2 and SUNE1), the activities of Caspase-3 was detected. The results were showed that, compared with the LNA-controls, the relative activity of Caspase-3 increased by (3.00±0.05) fold in the CNE2 cells of LNA-antimiR-214 group (*P*<0.001, Figure 2B). Similarly, compared with the LNA-controls, the relative activity of Caspase-3 increased by (2.73±0.09) fold in the SUNE1 cells of LNA-antimiR-214 group (*P*<0.001, Figure 2B).

Moreover, flow cytometry was also used to assess apoptosis in NPC cells transfected with LNA-antimiR-214 or LNA-control. Significant differences of Annexin-V-positive apoptotic cells were observed in the LNA-antimiR-214-treated group in comparison to cells transfected with LAN-control. LNA-antimiR-214 and LAN-control induced apoptosis in 23.75%±0.21% and 3.1% of CNE2 cells, respectively (*P*<0.001, Figure 2C). In SUNE1 cells, LNA-antimiR-214 and LAN-control induced apoptosis in 18.97%±2.21% and 1.97%±0.32%, respectively (*P*<0.001, Figure 2C). These results suggest that miR-214 inhibition can induce apoptosis in CNE2 and SUNE1 cells.

### Knockdown of miR-214 results in cell growth suppression

**Figure 3:**
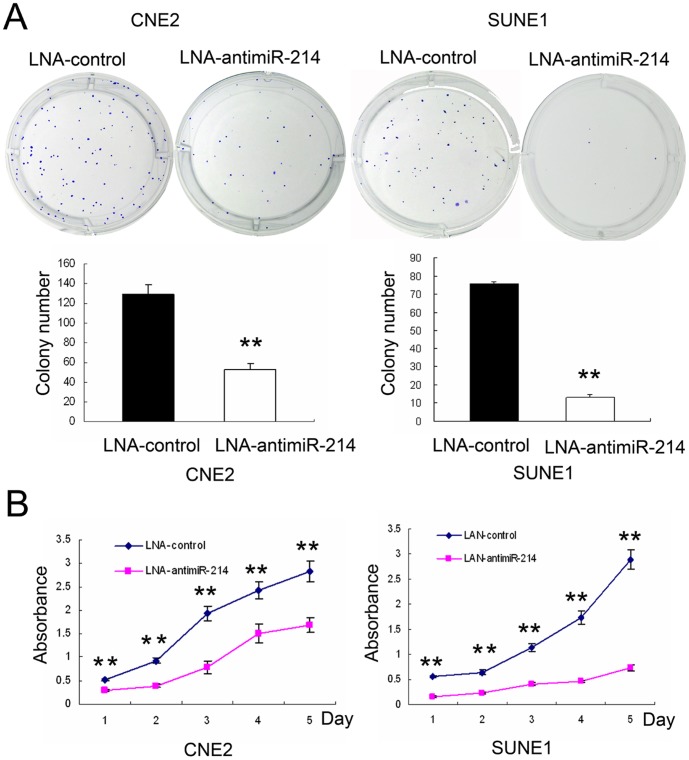
Silencing of miR-214 suppresses cell proliferation in NPC cells. (A) Colony formation assay. Twenty-four hours post-transfection, CNE2 and SUNE1 cells were seeded into 6-well plates with complete medium and incubated at 37°C for 2 weeks. (B) MTT assay. Twenty-four hours post-transfection, CNE2 and SUNE1 cells were seeded into 96-well plates. The colony formation assay (A) and MTT assay (B) showed that knockdown of miR-214 in CNE2 and SUNE1 cells resulted in inhibition of cell growth *in vitro*. All data are shown as mean ± SD of triplicate experiments. **P<0.01.

To address the function of miR-214 in NPC carcinogenesis, an inhibitor-mediated knockdown approach was employed to suppress the expression of endogenous miR-214, and subsequently determine the effect on cell growth. Briefly, we determined the effect of miR-214 on tumor cell proliferation by colony forming assays (Figure 3A) and MTT (Figure 3B). The results showed that knockdown of miR-214 led to remarkable inhibition of cell growth and proliferation in both CNE2 and SUNE1 cells.

### Knockdown of miR-214 suppresses tumorigenesis *in vivo*

**Figure 4:**
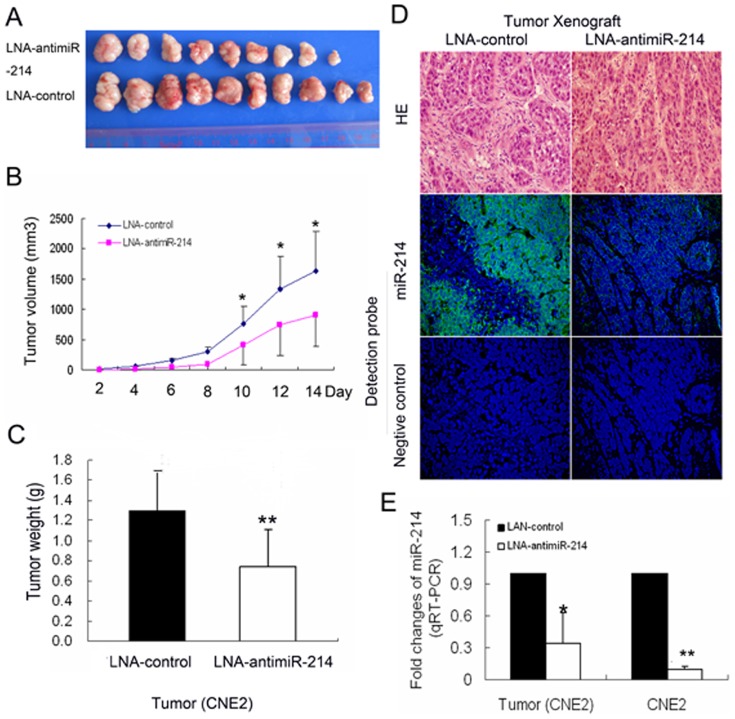
Knockdown of miR-214 inhibits tumor growth in nude mice: *in vivo* functional studies. (A) Photographs of tumors extracted from CNE2/LNA-antimiR-214 (n = 9) and CNE2/LNA-control groups. (B) Growth curves for CNE2/LNA-antimiR-214 (n = 9) vs. CNE2/LNA-control (n = 10) cells in an *in vivo* proliferation assay. (C) Tumors were weighed after animals were killed at 14 days post-tumor-cell injection. The weight of tumors was significantly decreased in CNE2/LNA-antimiR-214 group compared to CNE2/LNA-control group, (*P* = 0.006, Mann-Whitney test). (D) Representative photomicrographs of FISH for miR-214 on xenograft tumor sections obtained from mice bearing CNE2/LNA-antimiR-214 (n = 9) and CNE2/LNA-control groups (×400). (E) qRT-PCR analysis of miR-214 expression in CNE2 cells 48 h post post-transfection and in xenograft tumors after mice sacrifice. Data in (B), (C) and (E) indicate the mean ± SD. * *P*<0.05, ** *P*<0.01.

To address the potential effects of miR-214 on the growth of nasopharyngeal carcinoma cells *in vivo*, CNE2 cells transfected with LNA-antimiR-214 or LAN-control were subcutaneously injected into nude mice. As seen in Figure 4B, tumors rapidly formed in mice injected with CNE2 cells transfected with LAN-control, but injection of cells transfected with LNA-antimiR-214 lead to much lower tumorigenicity (All *P* values<0.05, Mann-Whitney test). Similarly, compared to the LAN-control group (10/10, 1.3 g±0.39 g, mean ± standard deviation), mice injected with LNA-antimiR-214 -transfected cells (9/10, 0.74 g±0.36 g, mean ± standard deviation), showed a significant decrease in both tumor number and weight of CNE2 (*P* = 0.006, Mann-Whitney test) (Figure 4C). MiR-214 expression in xenograft tumors of LNA-control group was higher than that of the LNA-antimiR-214 group (*P* = 0.017; Figure 4D, 4F).

### Bim is the target gene of miR-214

**Figure 5:**
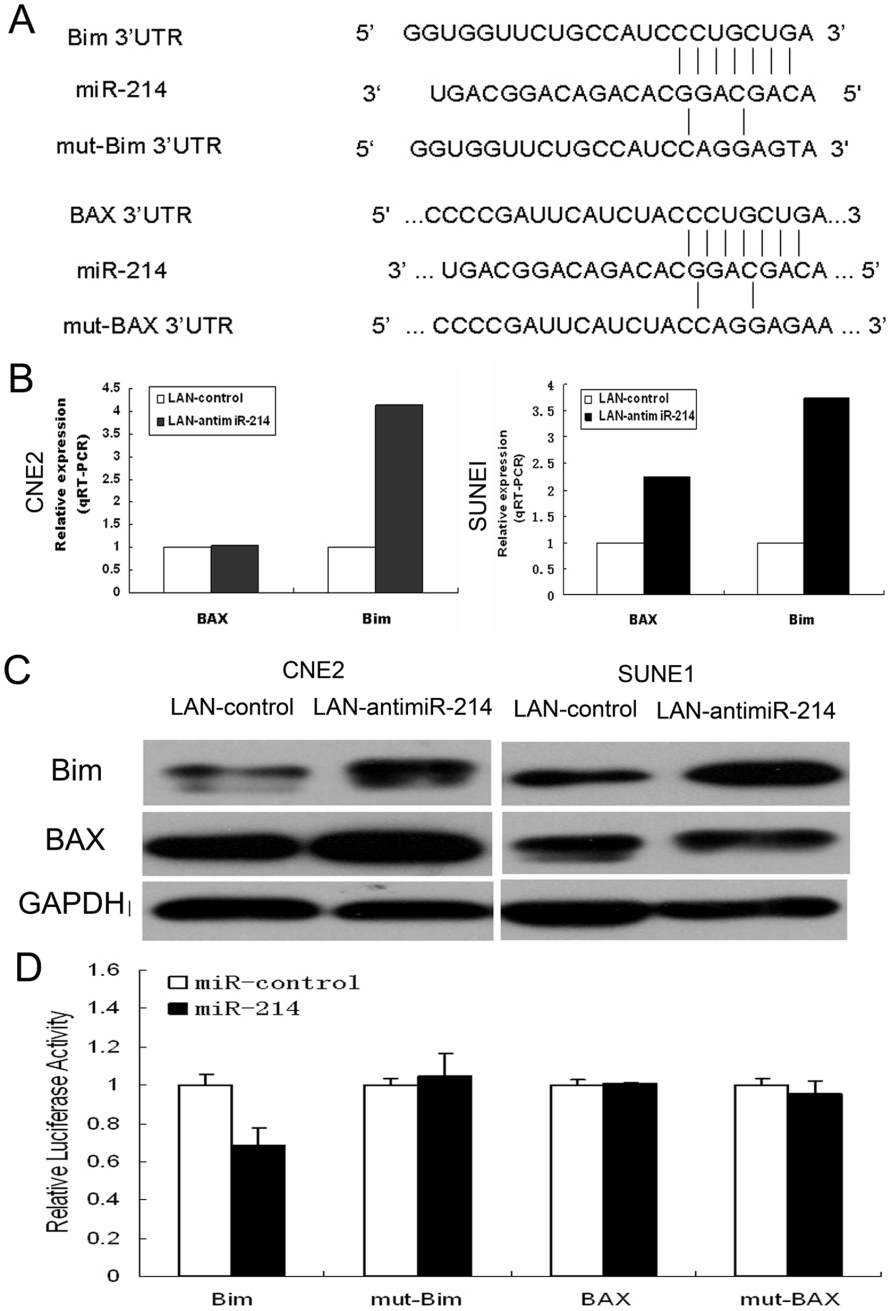
Bim is a direct target of miR-214 in NPC cell lines. (A). Pairing of miR-214 with Bim 3′UTR region and BAX 3′UTR region. (B and C). qRT-PCR and Western blot showed that knockdown of miR-214 using LNA-antimiR-214 led to upregulation of endogenous Bim and BAX in both CNE2 and SUNE1 cells, respectively. (D) Overexpression of miR-214 by the miR-214 mimic resulted in a significant decrease in luciferase signals of pMIRreport- Bim 3′UTR transfected 293T cells, but not in pMIRreport- BAX 3′UTR transfected 293T cells.

We predicted potential direct targets of miR-214 by at least three of TargetScan6.2, PicTar5, miRWalk, miRDB, DIANAmT and miRanda 3.0 programs. Two genes (Bim and BAX) were predicted to have at least one potential binding site at their 3′-UTRs for miR-214 (Figure 5A). We further investigated the effect of miR-214 on the expression of Bim and BAX by qRT-PCR as well as Western blot. Obviously, the result showed that LNA-antimiR-214 led to upregulation of Bim and BAX in both CNE2 and SUNE1 cells (Figure 5B–C).

We further tested whether miR-214 could directly repress the identified mRNA targets through 3′ UTR interactions (Figure 5A). Thus, the full-length 3′ UTRs of the human genes Bim and BAX were cloned into the downstream of the luciferase gene (pMIR-REPORT), respectively. These plasmids and pCMV-Renilla (internal control) and miR-214 or miR-control were transiently transfected into 293T cells. 48 hours after transfection, a dual-luciferase reporter assay system was used to detect luciferase expression. Overexpression of miR-214 resulted in a significant decrease in luciferase expression in pMIR-report-Bim 3′UTR-transfected cells, but not in pMIR-report-mut-Bim 3′UTR-transfected cells, compared with the miR-control. However, transfection of pMIR-report-BAX 3′UTR and pMIR-report-mut- BAX 3′UTR did not display significant reduction of luciferase levels (Figure 5D). In brief, these results indicated that Bim was a direct target of miR-214.

### Correlation between Bim expression and clinicopathological outcomes of NPC

**Figure 6:**
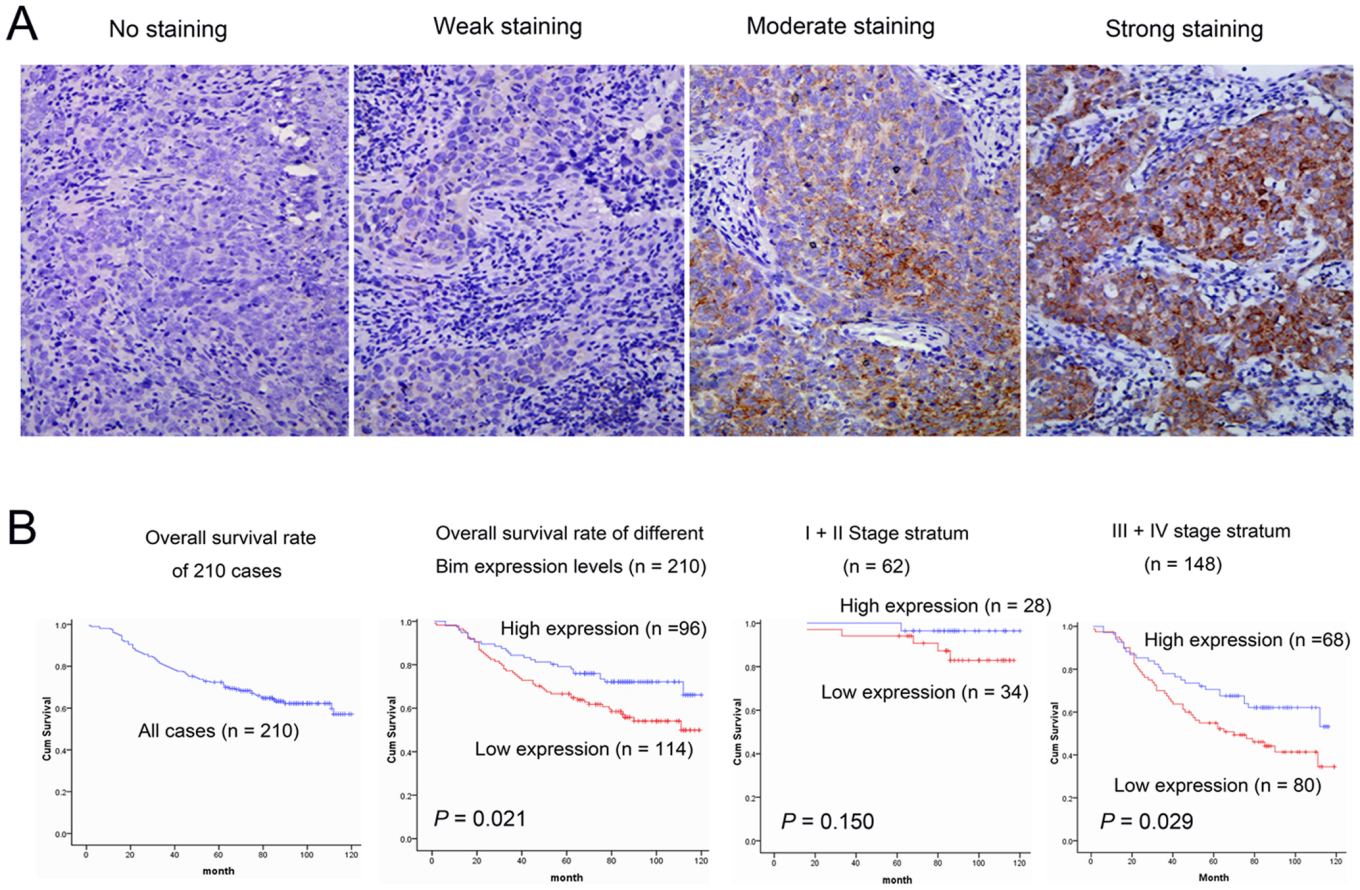
Bim expression and survival in NPC patients. (A) Representative staining of Bim in the cytoplasm of NPC cells, by immunohistochemistry at 200× magnifications. (B) Kaplan-Meier estimates of overall survival curves for different Bim expression levels in NPC patients, stratified by clinical stage.

To investigate Bim protein expression in NPC, IHC was carried out to detect Bim expression in NPC tissues. Bim was found predominantly in the cytoplasm of NPC tumor cells (Figure 6A). No significant correlation was observed between Bim and clinicopathological parameters (Table S1). The 5-year overall survival rate of the 210 NPC patients was 70.95%. The 5-year overall survival rate of patients with low Bim expression (56.14%, n = 114) was significantly lower than those with high Bim expression (78.13%, n = 96; *P* = 0.021, Figure 6B). Further analysis was performed in regard to Bim expression in subsets of NPC patients in different clinical stages. The results demonstrated that low Bim expression was also a prognostic factor in NPC patients with stage III and IV (*P* = 0.029), but not in NPC patients with stage I and II (*P* = 0.15) (Figure 6B).

## Discussion

The increasing evidence has been shown to be aberrant expression of some miRNAs in various types of cancers, and the alteration of miRNA expressions might lead to human carcinogenesis by negatively regulating multiple target genes [22]. MiR-214 is upregulated in several human tumors, such as ovarian cancer, gastric cancer, Sezary syndrome, and melanoma [10], [11], [12], [13]. Furthermore, elevated expression of miR-214 was associated with chemotherapy resistance [21] or tumor metastasis [13]. However, miR-214 expression was reduced in cervical cancer [14], [15], [16], pancreatic cancer [17], hepatocellular carcinoma [18], [19] and breast cancer [20], suggesting a tumor suppressor gene-like function. In this study, miR-214 was identified to be significantly higher expression in NPC compared to control. This was consistent with Dr. Li and his colleagues' study showing high-level of miR-214 in NPC [23]. In addition, knockdown of miR-214 in NPC cells could increase apoptosis and suppress cell growth, proliferation *in vitro* and suppress tumour growth *in vivo*.

That miRNAs regulating various biological processes suggests each miRNA may regulate hundreds of gene targets [3]. At present, several targeted genes of miR-214 have been identified in various tumor types. Previous study reported that miR-214 induces cell survival and cisplatin resistance of ovarian cancer primarily through targeting the PTEN [21]. MiR-214 has also been identified to be involvement of cervical cancer development by targeting MEK3, JNK1 [14], GALNT7 [15] and Plexin-B1 [16]. MiR-214 could contribute to melanoma tumour progression through suppression of TFAP2C. Moreover, some other genes implicated in cell proliferation, invasion and apoptosis, were direct targets of miR-214, including Ezh2 [20], HDGF [19], Lactoferrin [24], and so on. In our study, we found that knockdown of miR-214 could increase endogenous Bim protein expression in NPC cells (CNE2 and SUNE1). Luciferase reporter assay was performed to identify Bim as a direct target of miR-214 in NPC cells. Compared to Dr. Li and his colleagues' study, lactotransferrin was identified as a target gene of miR-214 [23]. It was concluded that miR-214 acted as different biological function through different target gene in NPC. Similar to other genes, the complexity of miR-214 biological function relys on different downstream signals that would play a pivotal role in the NPC tumorigenesis and been further investigated. Additionally, low Bim expression in NPC tissues was correlated with poor survival of NPC patients in our study. These findings suggested that miR-214 could promote NPC progression due to partially repressing endogenous Bim.

Bim is a BH3-only protein (BOP), a pro-apoptotic member of the Bcl-2 protein family and its expression is regulated both transcriptionally and posttranslationally [25], [26]. The forkhead-like transcription factor FOXO3a (Forkhead box O 3a) [27], [28] and tumor suppressor gene Runx3 (Runt-related protein 3) [29] are key transcriptional regulators of Bim. The other important regulation of Bim is the posttranslational regulation that includes phosphorylation and ubiquitination. For example, ERK [30], [31], p38MAPK [32], JNK [33], [34]-mediated phosphorylation of Bim affect the expression level or the proapoptotic function of Bim. In addition, ERK [35] and Caspase-3 [36] modulate Bim function via the ubiquitin. Therefore, Bim has played an important role in regulating cell proliferation and apoptosis. There are several studies focusing on the relationship between Bim expression and carcinogenesis and therapeutic strategies [37], [38], [39]. Previous study reported that co-treatment with TNF-α-neutralizing antibody and melphalan induced enhanced expression of Bim in multiple myeloma cell lines [40]. In Hela cells, Bim expression was enhanced during the treatment of 4-(3′,3′-Dimethylallyloxy)- 5-methyl-6-methoxy-phthalide (DMMP) to induce the apoptosis that would be a potential therapy for cervical cancer [41].

Even though interesting and rational, there are some limitations in this study. In order to completely understand the role of miR-214 in clinical practice, the levels of miR-214 in the FFPEs sample would be analyzed to determine whether miR-214 is a hallmark for prognosis and diagnosis. With respect to the relationship of miR-214 and Bim, further studies on knockdown of Bim in vivo and in vitro might give us more information for better understanding the roles of Bim expression affected by miR-214 on proliferation and apoptosis in nasopharyngeal carcinoma.

In conclusion, we report that miR-214 expression was increased in NPC tissues and NPC cells. Knockdown of miR-214 could suppress cell proliferation *in vitro* and *in vivo*, and induced apoptosis *in vivo*. Our findings suggest that miR-214 could play an important role in NPC carcinogenesis, and might be a better way of being prognostic or potentially therapeutic target for NPC patients.

## Materials and Methods

### Ethics statement

All the human tissue samples for routine pathology use were obtained from the Department of Pathology, Sun Yat-sen University Cancer Center (SYSUCC). For adults, written informed consent was obtained, while for children both verbal assent from the children and written consent were obtained from a parent or their legal guardians according to the declaration of Helsinki and experimentation. This study was approved by the Medical Research Ethics Committee of SYSUCC, and Animal Care and Use Committee.

### Patients and tissue specimens

The 210 NPC cases were selected randomly from January 2000 to December 2002 at SYSUCC and made into TMAs using a MiniCore Tissue Arrayer (Alphelys, Plaisir, France) with a 1-mm needle. The disease stage of all patients were classified according to the China 1992 NPC TNM staging system [42]. The clinicopathological characteristics of 210 NPC patients are given in Table 1. For the purpose of investigating the endogenous expression of miR-214 in NPC tissues, 5 NPC patients and 5 HCs were enrolled randomly between January 2009 and December 2009 at SYSUCC.

**Table 1:** Clinicopathological characteristics and follow-up data of 210 NPC patients.

Characteristic	Number of patients (%)
Sex	
Female	65 (31.1)
Male	144 (68.9)
Missing	1
Age (years)	
Median (range)	47 (17–78)
Follow-up time (months)	
Median (range)	81 (1–120)
Clinical stage	
I–II	62 (29.8)
III–IV	146 (70.2)
Missing	2
Recurrence	
Yes	15 (7.1)
No	195 (92.9)
Metastasis	
Yes	22 (10.5)
No	188 (89.5)
Therapeutic modality	
Radiotherapy	181 (86.2)
Radiochemotherapy	29 (13.8)
WHO histological classification	
NKUC	167 (80.0)
NKDC	38(18.2)
KSCC	4 (1.8)
Missing	1
OS rate (%)	
5-year	72.3

Abbreviations: NPC, nasopharyngeal carcinoma; WHO, World Health Organization; NKUC, non-keratinizing undifferentiated carcinoma; NKDC, non-keratinizing differentiated carcinoma; KSCC, keratinizing squamous cell carcinoma; OS, overall survival.

### Cell culture and knockdown of miR-214

The NPC cell lines (CNE2, SUNE1 and HONE1) were purchased from American Type Culture Collection (ATCC) (ATCC, Manassas, USA) and used within two months after resuscitation of frozen aliquots. The immortalized nasopharyngeal epithelial cell (NPEC2 Bmi-1) [42] was kindly provided by Prof. Mu-Sheng Zeng. The NPC cell lines (CNE2, SUNE1 and HONE1) were cultured in RPMI 1640 with 10% fetal bovine serum (Invitrogen, CA, USA). The immortalized nasopharyngeal epithelial cell, NPEC2 Bmi-1 was cultured in KSF (Invitrogen, CA, USA). All the cell lines were grown in a humidified incubator at 37°C with 5% CO_2_.

CNE2 and SUNE1 cells were plated in 6-well dishes at 2×10^5^ cells/well. Knockdown experiments were done after 24 hours seeding. LNA-control or LNA-antimiR-214 (Exiqon A/S, Vedbaek, Denmark) 50 nmol were transfected into cells using reagent of Lipofectamine RNAiMAX (Invitrogen, CA, USA) according to the manufacturer's instruction.

LNA-antimiR-214 sequence was 5′-TGCCTGTCTGTGCCTGCTG-3′.

### Mature miRNA quantitative RT-PCR (qRT-PCR)

Total RNA was extracted from cell lines and FFPE tissue using Trizol reagent (Invitrogen). We synthesized cDNA using One Step PrimeScript^???^ miRNA cDNA Synthesis Kit (TaKaRa). The expression of mature miR-214 was determined by SYBR Green quantitative real time PCR amplification on an ABI 7500HT instrument (Applied Biosystems). For data analysis, cycle threshold (*Ct*) for U6 (reference) and miR-214 (sample) were determined in triplicate (shown as arithmetical mean). The relative expression was calculated using the relative quantification (RQ) = 2-ΔΔ*^Ct^* [43], in which ΔΔ*C_T_* = (mean *C_T_*_ cancer_ – mean *C_T_*_ control_) - (mean *C_T_*_ health_ – mean *C_T_*_ control_).

### Western blot analysis

Cells were lysed in SDS lysis buffer and incubated for 10 minutes at 95°C. 50 ug of total cell lysates per lane was separated by 10% SDS-PAGE. Antibodies used for immunoblot analysis were against Bim with 1∶1000 dilutions, BAX with 1∶1000 dilutions (Cell Signaling Technology, Shanghai, China) and GAPDH with 1∶2000 dilution (Santa Cruz Biotechnology, CA, USA) as a loading control.

### Tyramide Signal Amplification (TSA) for FISH

Fluorescence in situ hybridization (FISH) of miR-214 was performed on 5 µm tissue sections of NPC following the manufacturer's instructions. Briefly, sections were at 59°C 2 h to attach cores to the silane-coated slide. Then, sections were de-paraffinised with xylene two times for 5 minutes each, rehydrated with ethanol (100 - 50 - 25% for 5 min each), and treated with DEPC water for 1 min. Subsequently, sections were treated with pepsin solution (1.3 mg/ml) (Dako, Glostrup, Denmark) at 37°C for 30 min. Following a post-fixation step in 4% paraformaldehyde (PFA), LNA-miR-214 detection probe (Exiqon, Vedbek, Denmark) or LNA-control (Exiqon, Vedbek, Denmark) was hybridized to the sections at 58°C for 4 hours carried out in a Hybrite (Abbott Laboratories, Shanghai, China). After post-hybridization wash, sections were incubated with anti-DIG-HRP (Roche, Shanghai, China) in a heater at 37°C for 30 min. Slides were washed by TNT buffer for 15 min at room temperature (RT) and incubated with Fluorophore TSA Reagent working solution for 10 min at RT. The slides were counterstained with 4′, 6-diamidino-2-phenylindole (DAPI) (Roche Diagnostics, Shanghai, China) and visualized the nuclei and glass mounting by fluorescence microscopy with a Jenoptik camera and VideoTesT-FISH 2.0 software (GP Medical Technologies Ltd, Beijing, China).

### Determination of Caspase-3 activity

For determination of Caspase-3 activity in the cells, Caspase-3 Colorimetric Assay kit (KeyGEN Biotech, China) was used according to the manufacturer's instructions. Briefly, cells (4×10^6^) in each group mixed with 50 µl cellular lysis buffer and then incubated on ice for 30 min. After centrifugation at 4°C for 5 min, the supernatant (50 µl) was collected and mixed with 50 µl 2× Reaction buffer and 5 µl Caspase-3 substrate. The complexes was incubated at 37°C in the dark for 4 h, the optical density values of the samples were read on a spectrophotometer at a wavelength of 405 nm, which represented the intracellular activity of caspase-3. All analyses were performed in triplicate.

### Apoptosis assay

For apoptosis analysis, cells were collected 48 hours post-transfection, and then stained with Annexin V-FITC (5 µl) and propidium iodide (5 µl) using the Annexin V-FITC Apoptosis Detection Kit (KEYGEN Co. Ltd., Guangzhou, China). The percentage of apoptotic cells was quantified using a Flow cytometry (Beckman Coulter Corp., CA, USA). All analyses were performed in triplicate.

### Cell proliferation assay and colony formation assay

For cell proliferation assay, 24 h post-transfection, the cells of each group were reseeded in 96-well plates at a density of 2×10^3^ cells/well, and incubated overnight in 150 µL culture medium. Twenty-four hours later, cells were stained with 20 µl 3-(4, 5-dimethylthiazol-2-yl)-2, 5-diphenyltetrazolium bromide (MTT) (5 mg/ml, Sigma, St. Louis, Missouri, USA), and incubated at 37°C for 4 h. After removal of the supernatant, 150 µl dimethyl sulphoxide (Sigma, Shanghai, China) was added and thoroughly mixed for 15 min. Absorbance was measured with a microplate reader of SpectraMax M5 (Molecular Devices Corp., CA, USA) at a wave length of 570 nm.

For colony formation assays, 24 h post-transfection, the cells of each group were reseeded in 6-well plates (200 cells per well) and cultured at 37°C for two weeks. Colonies were fixed with methanol for 10 min, and stained with 1% crystal violet (Sigma) for 20 min. All analyses were performed in triplicate.

### The prediction of potential targets of miR-214

TargetScan6.2, PicTar5, miRWalk, miRDB, DIANAmT and miRanda 3.0 programs were used to predict putative targets of miR-214 respectively. Bim and BAX (BCL2-associated X protein) were predicted by at least three programs and selected for further validation.

### Luciferase reporter assay

Bim, mut-Bim, BAX and mut-BAX were cloned into pMD19-T by Simple TaKaRa Biotechnology and then individually subcloned downstream to the luciferase coding sequence in the luciferase reporter pMIR-Report-Vector (Life Technologies, Beijing, China). For Luciferase reporter assays, 293T cells were seeded in 12-well plates and transfected with wide type or mutant reporter constructs (50 ng) together with miR-214 or miR-control (50 nM, GenePharma, Shanghai, China) and Renilla plasmid (10 ng) using lipofectamine 2000 (Invitrogen, Carlsbad, CA, USA). 48 h post-transfection, the cells were lysed and luciferase activity was measured by using Dual Luciferase Assay (Promega). Firefly luciferase values have been normalized to Renilla, and the ratio of firefly/renilla was presented.

### Immunohistochemical staining

Immunohistochemistry staining was performed to examine the expression of Bim in NPC tissues. Primary antibody against Bim with 1∶500 dilution (Cell Signaling Technology) was used in this study. Three observers determined consensus scoring of Bim immunostaining using a semi-quantitative estimation independently [44]. Briefly, tumor cell staining was assigned a score using a semiquantitative four-category grading system: 0 = 0–5% of tumor cells stained; 1 = 6–25% of tumor cells stained; 2 = 26–50% of tumor cells stained; 3 = more than 50% of tumor cells stained. Likewise, staining intensity was assigned a score as follows: 0 = no staining; 1 = weak staining; 2 = moderate staining; 3 = strong staining. Cases with scores less than and equal or more than the median value were considered as low expression or high expression.

### Validation of tumor growth-promoting activity of miR-214 in an animal model

Female BALB/c-nude mice (Hunan Slac Jingda Laboratory Animal Co., Ltd., Hunan, China) aged 4 to 5 weeks, were used for tumor xenografts. The nude mice were then randomly divided into two groups, CNE2-LNA-control group and CNE2-LNA-antimiR-214 group, respectively. CNE2 cells treated with LNA-antimiR-214/LNA-control (100 nM for 24 h) were injected subcutaneously (1×10^6^ cells/tumor) into the left axilla of nude mice in the CNE2-LNA-antimiR-214 and CNE2-LNA-control groups. Tumor width (W) and length (L) were measured every day. Mice were killed 14 days post-injection, and tumors from the two groups were extracted and weighed. Tumor volume was calculated according to the standard formula: V = Π/6×L×W^2^. All experiments were in accordance with the Ethics Committee for Animal Research of SYSUCC (Reference number: S10211041).

### Statistical Analysis

Data was analyzed using SPSS16.0 software. Survival curves were estimated by Kaplan-Meier analysis and compared by the log-rank test. Data were expressed as mean ± SD, and T-test was used to determine the significance of differences in multiple comparisons. All tests performed were two-sided. P<0.05 was considered statistically significant.

## Supporting Information

Table S1 (XLS)
